# Vaccination and Compulsory Vaccination

**Published:** 1894-09-15

**Authors:** 


### The Hospital

An Institutional Journal of

The Medical Sciences and Hospital Administration, with

AN EXTRA NURSING SUPPLEMENT.

Vol. XVI.—No. 416.] [Sept. 15, 1894.

### Vaccination and Compulsory Vaccination.

Last Friday more than two whole columns of the *Times* were occupied by the two questions of vaccination and compulsory vaccination. For these two, it is to be remembered, are entirely distinct questions. For many days previously there had been in the same newspaper intermittent letters on both sides of the compulsion argument. Compulsory vaccination is, indeed, a burning question of the day, like the Eight Hours Bill or Home Rule for Ireland. It is a doctor's question; not a question merely for the doctor's science, but for the doctor's statesmanship. Assuming, as we do, that vaccination has all the scientific, all the prophylactic, all the social value which has ever been claimed for it, we have still to consider whether in the region of high politics the medical profession is acting wisely in assuming the entire responsibility for universal compulsion.

If, as is believed, Dr. Klein and Dr. Simpson, of Calcutta, have actually established the identity of cow-pox with small-pox, the discussion is advanced by more than a step. There seems to be no reason to doubt that Dr. Klein and Dr. Simpson have really established the identity of these two affections. Those who wish to study the researches of these investigators in detail should procure the recently-issued report of the Medical Officer of the Local Government Board. But the long stride by which Dr. Klein and Dr. Simpson have advanced the discussion of the vaccination question is not necessarily a stride in the direction of compulsion. It is a stride in the direction of definite scientific knowledge where before there was a most unscientific vagueness, and a stride in the direction of full experimental assurance in the medical mind where before there was mainly or only the assurance of high probability and statistics. But, as even the most determined of compulsionists is now beginning to see, there is all the difference in the world between the establishment of the value of vaccination as a prophylactic against small pox and the insistence upon universal compulsory vaccination at the point of the legal sword.

The present generation of medical men have inherited compulsory vaccination from their professional ancestors. But is that of itself a sufficient reason why they should stand by it as an essential article of the faith of their medical politics? The present generation believes firmly in the prophylactic value of vaccination; but because it believes it has patience to wait for the universal voluntary acceptance of demonstrated truth. There are other diseases, such, for example, as cholera, which assume the proportions of devastating epidemics, and which, in the case of cholera at any rate, there is every reason to believe may be prevented by scientific methods of prophylaxis. Indeed, the prospect which now looms large before the medical mind is that all bacillary diseases will become controllable by prophylactic inoculations. But if the medical mind should once be satisfied that prophylactic inoculations are preservatives against cholera, diphtheria, typhoid fever, scarlet fever, and the like, does it follow that the medical profession is to insist upon universal compulsory inoculations for the prevention of all these diseases? As a matter of strict logic and consistent state policy it may follow that if vaccination against small pox is always and everywhere to be made compulsory, then the demonstration of such prophylactic value as will satisfy the medical mind in the case of cholera and other bacillary inoculations should be the signal for universal compulsion here also. But the medical profession may well pause and consider its position before committing itself to the very grave responsibility of advising the State to make compulsion universal in all these cases.

This is the position whichwe as a profession have to face; and we shall probably never have so favourable an opportunity for facing it as now. We have come to the parting of the ways between the new and the old. All the world gives us credit for honesty of purpose and for high scientific competence. Why should we not win for ourselves as individuals and for our great calling that other and equal claim to honour which a reasonable and right-minded judgment in public policy will entitle us to make? The world will honour us if we make cures; it will honour us also if we prevent diseases; it will honour us most of all if in all our methods we treat it with the profound respect which is always and everywhere due to the reasoning mind. No man of any self-respect likes to be driven like a bullock to the market, even though his arrival at the market should be for his own undoubted good. The history of compulsion in religion, a compulsion which has always failed with the intellectual, may well make physiologists and physicians think many times before they finally and irrevocably commit themselves to a policy which, however legitimate it may be and is on the part of the State, is not, by any means, among the most honoured methods of scientific progress.

There are better ways than naked compulsion; there is the way of example, there is the way of instruction. We who believe in vaccination can vaccinate ourselves, our children, and all over whom we can exert a reasonable influence. Moreover, we can instruct. By tongue and pen we can persuade the world of our scientific competence , and at the same time of the honesty of our purposes. Is not this the very time for the beginning of such a new and more effective policy? What, after all, should be the purpose of the medical profession in this matter? Not, surely, the mere bending of an obdurate public to the medical mind and will by the use of the sword, but the enlightenment, the convincing of the public mind by the employment of the infinitely nobler methods of patient culture and persuasive mental illumination. The late Archbishop Magee once said on a memorable occasion that "he would rather see Britain free than sober." Therein, by a paradox, he expressed a political conviction of the noblest and most elevating kind. As a matter of fact, the freer Britain has grown the soberer she has become. So will it be with questions of medical prophylaxis. Let the people be instructed, let their minds be convinced, and then they will promptly yield to that most absolute of all compulsions, the inner compulsion of a willing and grateful heart. In all this our purpose is not to advocate change in the vaccination laws, because no such change is demanded in the public interests. It is to ask our fellow medical men, and the profession as a whole, to take up towards the public the legitimate scientific position of persuasion and instruction, and thereby to convince the general reason that compulsion, for which the State and not the medical profession is responsible, is in the case of vaccination against small-pox, at any rate, a proved necessity. If this conviction be established, the pathway of the scientific inoculator for other bacilliary diseases will be greatly smoothed, and gratitude, rather than hatred and opposition, will be the reward of our scientific and self-denying profession.

